# Mechanistic Links Between DNA Methylation and Protein Translation and Their Impacts on Brain Development

**DOI:** 10.3390/biology15090687

**Published:** 2026-04-28

**Authors:** Ashraf Kadar Shahib, Mojgan Rastegar

**Affiliations:** Department of Biochemistry and Medical Genetics, Max Rady College of Medicine, Rady Faculty of Health Sciences, University of Manitoba, Winnipeg, MB R3E 0J9, Canada

**Keywords:** brain development, DNA methylation, epigenetics, protein translation, mTOR signaling, neurodevelopment, ribosome biogenesis, epigenetic mechanisms, MeCP2

## Abstract

This article explores the complex interplay between the process of protein translation and DNA methylation, discussing their combined involvement in brain development. We will emphasize on DNA methylation and related proteins such as DNMTs, TETs, and MeCP2, the latter being the prototype of DNA methyl-binding proteins. Collectively, DNA methylation machinery may be involved in controlling the cell fate commitment of brain cells, as well as their neuronal and glial lineage specification. We aim to summarize current knowledge on the dynamics of protein translation, ribosome biogenesis, and relevant cellular pathways, including the mTOR signaling, in the context of brain development. Special attention is given to MeCP2 because of its unique role as an epigenetic factor that influences the chromatin states with a link to protein translation and its relevance to human disease. We also discuss the impact of DNA methylation-mediated chromatin regulation and protein translation in neurodevelopmental disorders. Our discussions include multi-omics techniques and integrative mechanisms that connect DNA methylation with protein translation.

## Simple Summary

The process of protein translation is tightly controlled to support fundamental brain function such as learning, memory formation, and behavior. This review focuses on the interplay between protein translation machinery and DNA methylation in the main brain cell types. We describe key epigenetic factors that include the writers, readers, and erasers of DNA methylation and how neurons regulate protein synthesis through signaling pathways, responsive to neuronal activity and cellular metabolism. Our focus is on the epigenetic reader “MeCP2” that bridges DNA methylation and protein translation initiation. MeCP2 is a multifunctional nuclear protein, and its genetic mutation causes Rett Syndrome. Our review aims to highlight mechanistic links that may have been overlooked in the field, emphasizing neuronal-specific gene regulation and potentially future therapeutic promises.

## 1. Introduction

Epigenetic mechanisms are heavily involved in the regulation of brain development, bridging extrinsic environmental stimuli and internal genetic cues to precisely control gene expression. From the earliest phases of embryonic development to postnatal growth and adulthood, these intricate regulatory networks coordinate the process of neurodevelopment. The timely and coordinated changes in gene activity are very important for the proper formation of neuronal differentiation, maturation, and synaptic plasticity. Accordingly, the brain is vulnerable to abnormalities in these epigenetic pathways that can be influenced by environmental factors. In addition, the dynamic remodeling of cellular epigenetic landscapes during brain development and cellular differentiation is studied in the context of DNA methylation, histone post-translational modifications (PTMs), and regulatory non-coding RNA molecules, collectively recognized as important key regulators of neurodevelopment [1,2,3,4].

An important epigenetic modification that controls gene transcription, which is also involved in chromatin compaction, is “DNA methylation”. DNA methylation refers to the covalent addition of methyl groups, mostly to the cytosine residues of DNA molecules. In brain cells, this modification is suggested to either stabilize the silencing of specific genes, or in contrast, promote context-dependent gene activation. Such diverse functional roles may depend on the type of DNA methylation. Concurrently, the control of how a transcript translates into a protein provides an additional regulatory layer of gene expression, which is crucial for adjusting protein synthesis as a response to developmental stimuli and brain activity. For neurons to react to synaptic demands and environmental stimuli, these translational processes and support the localized and temporally constrained protein synthesis [5,6,7,8,9].

According to recent research, DNA methylation and protein translation work hand-in-hand to influence brain plasticity and developmental trajectory rather than acting independently of each other. The overall protein content of a cell is controlled by the interplay between epigenetics (such as DNA methylation and histone modifications) and protein translation machinery. The methylation status of a transcript can affect its stability, subcellular localization, and activity and the translation of the encoded protein. Translational regulators may affect DNA methylation patterns by providing regulatory feedback on specific epigenetic enzymes. Determining the molecular foundations of brain development and treatment of neurological conditions that may result from deregulation of epigenetic mechanisms and/or translational processes depends on understanding these interactions [6,10,11]. With a clear focus on molecular mechanisms and their impairments in neurodevelopmental disorders, we aim to provide a thorough discussion of these topics.

## 2. Epigenetics and DNA Methylation in Brain Development

### 2.1. Epigenetic Mechanisms: The Role of Epigenetic Writers, Readers, and Erasers

Regulatory mechanisms that control gene expression and/or activity without any change in the order of underlying nucleotide sequences are considered as “epigenetics”. Cellular memories that control gene expression programs essential for brain development are tightly controlled by epigenetics. There are three main types of epigenetic factors, the writers, readers, and erasers, which in harmony orchestrate epigenetic regulation. These proteins write, erase, or interpret epigenetic marks, respectively. Examples of epigenetic writers include histone acetyl transferases, histone methyl transferases (SET-domain proteins), and DNA methyl transferases (DNMT1, DNMT3A and DNMT3B). On the other hand, erasers of DNA methylation are known as TET (Ten–Eleven Translocation) proteins. They modify 5-methylcytosine (5-mC) into 5-hydroxymethylcytosine (5-hmC), contributing in active DNA demethylation pathways, which are essential for gene activation. Histone demethylases and deacetylases can functionally remove the histone modifications to increase chromatin accessibility [3,8,9,12,13].

Epigenetic readers contain specific protein domains such as bromodomains or the methyl-CpG-binding domains (MBD). These factors identify particular epigenetic modifications and attract chromatin remodelers or transcriptional regulators depending on their interacting partners. The canonical epigenetic reader methyl-CpG-binding protein 2 (MeCP2) can either activate or repress its target genes by binding to methylated CpG sites [14,15,16]. The crucial role that epigenetic readers play during neurodevelopment is demonstrated by the fact that *MECP2* mutation is connected to a neurodevelopmental disorder known as Rett Syndrome (RTT), a severe and rare neurological disorder in young females [15,17]. The intricate interactions between the epigenetic readers, writers, and erasers create a dynamic multilayer regulatory system that supports synaptic activity and cell fate commitment in the brain [9,11].

### 2.2. DNA Methylation: 5-Methylcytosine (5-mC) and 5-Hydroxymethylcytosine (5-hmC)

The cytosine residues in CpG dinucleotides are the primary site for DNA methylation to happen, which transforms them into 5-mC, a characteristic of repressive/silenced chromatin state linked to inactive genes. However, non-CpG-methylation (CpH, where H could be A, T, or C) is also common in the brain, especially in post-mitotic neurons, and is involved in regulating neuronal gene expression. A paradigm shift in our knowledge of DNA methylation dynamics was made possible by the discovery of 5-hmC, which is formed by the enzymatic activity of TET proteins. In addition to acting as a durable epigenetic mark that promotes transcriptional activation, 5-hmC is substantially enriched in the brain tissue and is also considered an intermediate modification in active DNA demethylation [4,7,8,9,11].

The 5-mC and 5-hmC DNA methylations can be found at the gene bodies, promoters, and enhancers with developmental stage- and cell type-specific patterns in the central nervous system (CNS), based on genome-wide mapping studies. During neurogenesis and synaptogenesis, these patterns of DNA methylation offer an additional layer of regulation that can impact RNA polymerase II kinetics, transcription factor binding and the three-dimensional structure of the chromatin. As a result, DNA methylation patterns are dynamically changed during embryonic development and brain cell differentiation/maturation, and in response to neuronal activity, demonstrating how flexible epigenetic regulation could be in controlling gene expression [9].

DNA methylation and non-coding RNAs form interconnected regulatory layers that shape brain development, rather than acting in isolation. DNA methylation at miRNA and lncRNA promoters may control their expression at the transcriptional level. For instance, methylation of the *miR-7b* locus and MeCP2-dependent recruitment of methyl-CpG binding proteins can establish feedback loops that link miRNA outputs to neuronal maturation. The *MECP2-BDNF-miR132* homeostatic feedback loop, which regulates neuronal activity-dependent gene expression, is an example of neuronal-enriched miRNAs like *miR-132*. Long non-coding RNAs (lncRNAs) can also function upstream of DNA methylation. For example, lncRNAs produced in the CNS, such as *Dali (DNMT1-Associated Long Intergenic RNA)*, bind DNMT1 and affect CpG island methylation at neurodevelopmental genes, thereby coordinating chromatin status with transcriptional programs in developing neurons [18,19,20,21].

### 2.3. DNA Methylation Machinery: DNMTs, TETs, and MBDs in Neurodevelopment

**Figure 1:**
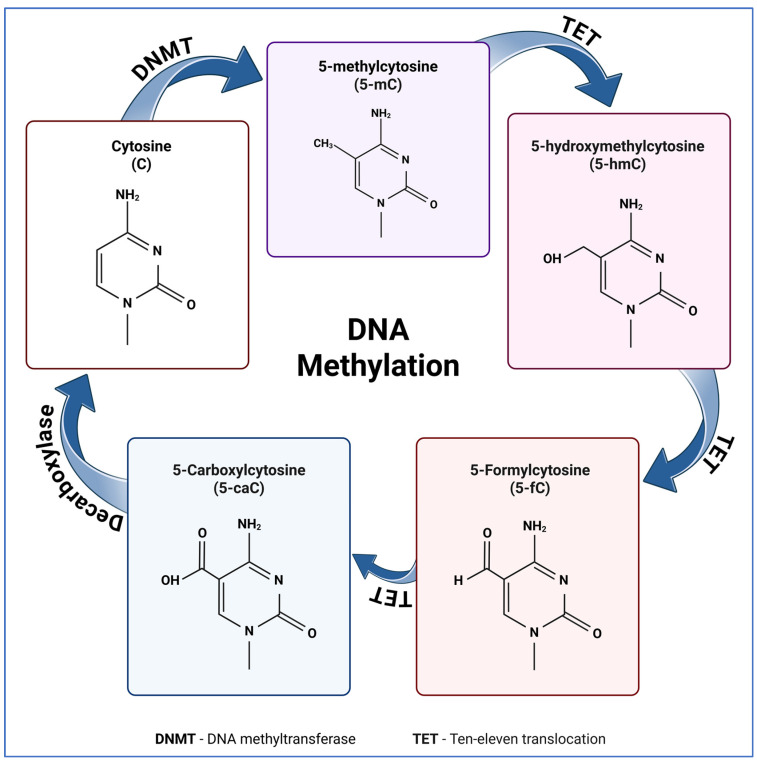
Schematic representation of dynamic DNA methylation. The cycle begins with the methylation of cytosine (C) at the carbon 5 position by DNA methyltransferases (DNMT) to form 5-methylcytosine (5-mC). This epigenetic mark can be actively changed through oxidative modification. The Ten-eleven translocation (TET) family of enzymes perform sequential oxidation of 5-mC into 5-hydroxymethylcytosine (5-hmC), 5-formylcytosine (5-fC), and 5-carboxylcytosine (5-caC). Figure adapted from [15] and generated by BioRender.

DNA methyltransferases are a group of enzymes that catalyze the deposition and maintenance of DNA methylation. The main function of DNMT1 is the maintenance of DNA methylation, which keeps DNA methylation patterns in dividing cells throughout DNA replication. The primary act of DNMT3A and DNMT3B is as de novo methyltransferases, catalyzing new DNA methylation in developing brain cells. The crucial function that DNMTs play in the differentiation, proliferation, and survival of brain cells is highlighted by the recent knockout and mutation studies conducted in mice. Through the sequential oxidation of 5-mC to 5-hmC and subsequent products (Figure 1), TET proteins, which include TET1, TET2, and TET3, perform active demethylation, mediating transcriptional regulation of target genes. Aberrant neuronal differentiation and neurodevelopmental problems may result from disruption of TET proteins [3,4,7,8,9].

Methyl-CpG-binding domain proteins such as MeCP2 binds to and interprets DNA methylation and bridge transcriptional control, chromatin remodeling, and DNA modifications. Rett Syndrome, the severe neurodevelopmental condition caused by mutations in MeCP2, demonstrates how incorrect epigenetic reading could interfere with brain development and function. Depending on the stage of development and cellular environment, additional MBD proteins (MBD1, MBD2, MBD3, and MBD4) would contribute to either transcriptional repression or activation. The epigenomic and transcriptomic progress necessary for brain development are coordinated by the balanced actions of DNMTs, TETs and MBD proteins [9,11].

### 2.4. Role of DNA Methylation in Brain Development

**Figure 2:**
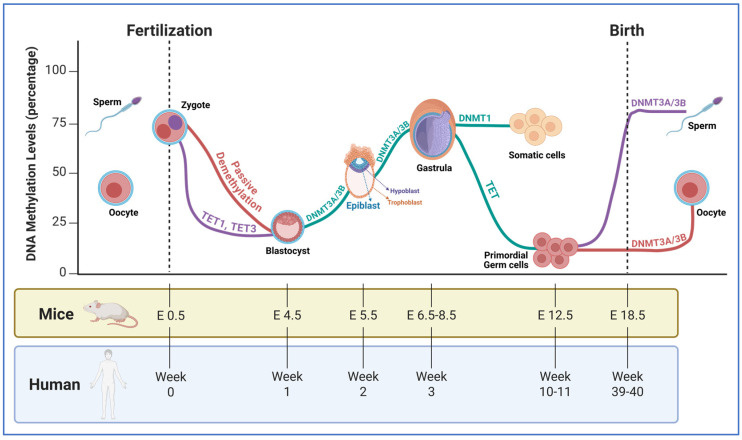
Dynamic global DNA methylation reprogramming during mammalian embryogenesis and germline specification. This schematic illustrates the fluctuations in global DNA methylation levels (y-axis, %) across the developmental timelines of both mice (embryonic days, E) and humans (weeks). The process is characterized by two major waves of demethylation and subsequent de novo methylation. Zygote to Blastocyst (First Wave)—After fertilization, the paternal genome (purple line) is subjected to rapid active demethylation facilitated by TET1 and TET3 proteins. On the other hand, the maternal genome (red line) undergoes gradual passive demethylation. By the blastocyst stage (E4.5/Week 1), the Inner Cell Mass (ICM) reaches a ground state of “naïve pluripotency” with minimal methylation. Lineage Commitment and Gastrulation: As the embryo progresses through the epiblast and gastrula stages, global methylation levels rise significantly. This de novo methylation is driven by DNMT3A/B enzymes, transitioning the cells from formative to “primed pluripotency.” DNMT1 subsequently maintains these patterns in somatic cells. Primordial Germ Cell (PGC) Specification (Second Wave): A subset of cells is specified as PGCs (approx. E12.5/Week 10–11). These cells undergo a second, nearly complete erasure of DNA methylation, mediated by TET enzymes, to reset the epigenetic clock and erase parental imprints. Sex-Specific Re-methylation: After a global DNA demethylation in the primordial germ cells, re-methylation of DNA molecules will be established based on biological sex. In the male germline (purple), DNA methylation increases rapidly before birth (E18.5/Week 39–40) during sperm development. In the female germline (red), methylation remains low through birth, only increasing during oocyte maturation in the postnatal period. Figure adapted from [23] and generated by BioRender.

Brain region-specific, cell type-specific, and temporally-specific DNA methylations that affect the process of neurogenesis, neuronal migration, synaptogenesis, and glial differentiation in part regulate the processes of brain development. Waves of DNA demethylation and re-methylation that correspond with significant developmental stages are characterized by genome-wide DNA methylation and hydroxymethylation studies (Figure 2). Because of variations in chromatin accessibility and transcription factor activity, neural stem cells, for instance, have different methylation profiles from differentiated neurons and glia. Hence, to promote lineage commitment, DNA methylation modification may occur in genes involved in neuronal destiny specification, including *NeuroD1* and *Pax6* [4,9,13,22].

Throughout early postnatal development till adulthood, genes linked to neuronal plasticity experience dynamic DNA methylation patterns that facilitate learning, memory, and synaptic refinement. A complex kind of epigenetic regulation is demonstrated by the fact that, whereas methylation at gene promoters often suppresses transcription, methylation at the gene bodies can correspond with active transcription. Stress, diet, alcohol exposure, and neuronal activity are examples of environmental factors that can further alter DNA methylation patterns during crucial periods of brain development, impacting cognitive function and susceptibility to disease [7,10,11,13]. During brain development, a number of internal and external stimuli dynamically affect DNA methylation, influencing circuit construction, neurogenesis, and neuronal maturation. Transcription factors like NeuroD1 and Pax6 are examples of intrinsic factors. In order to improve regional neuronal identity and stress response, steroid hormones such as 17β-estradiol and glucocorticoids block the transcription of hormone receptor promoters (ERα, GR) via inducing DNMT expression and methylation. Additionally, TET-mediated hydroxymethylation is driven by cell-intrinsic metabolic conditions, allowing demethylation waves that may trigger the activity of differentiation genes in post-mitotic neurons [24,25,26].

These DNA methylation patterns could be further modulated by extrinsic factors during important times. At plasticity genes like *Bdnf (Brain-derived neurotrophic factor)*, neuronal activity causes fast de novo methylation or TET1-dependent demethylation, which promotes synaptic refinement and memory formation. Through glucocorticoid surge or folate/one-carbon metabolism, maternal nutrition and prenatal stress could impact DNA methylation of the fetus, resulting in long-lasting hypomethylation of the hypothalamic stress axes and poor cognitive development. Alcohol and other environmental pollutants may also interfere with DNMT3A/B function, leading to neural tube abnormalities and worldwide hypomethylation. Together, these variables contribute to shaping DNA methylation landscapes, increasing vulnerability to neurodevelopmental diseases [1,26,27,28].

### 2.5. Role of DNA Methylation in Aging

A mechanistic role of epigenetic drift in aging is supported by DNA methylation, which exhibits distinctive age-associated alterations at CpG sites and may include global hypomethylation with concurrent regional hypermethylation at promoters and regulatory regions. Change in DNA methylation patterns is linked to functional decline and age-related diseases in coordination with these age-related methylation patterns, which can impact genes involved in development, cell cycle regulation, and stress responses [29,30].

The development of multivariate “epigenetic clocks,” in which weighted methylation levels at specific CpG panels accurately predict chronological age, was made possible by the systematic mapping of age-associated CpGs across different organs. Using 353 CpGs from various human tissues and cell types, Horvath’s pan-tissue clock established DNA methylation age as a reliable biomarker suitable for a variety of biological samples [31,32].

There is evidence of accelerating aging due to dysregulated DNA methylation patterns at specific loci that are linked to outcomes such as early death and/or cancer risk [33]. It has been suggested that DNA methylation could be considered as a quantitative biomarker to track aging and rejuvenation treatments [29,34,35].

## 3. Protein Translation

One fundamental biological process that is critical for cell function and growth is protein translation, producing proteins from messenger RNA (mRNA) templates. Precise translational control is essential for controlling neuronal differentiation, synapse formation, and plasticity in the brain, where intricate structural and functional modifications take place during neurodevelopment and life. In contrast to gene transcription, protein translation determines the proteome output in a geographically and temporally specific way, enabling quick, localized reactions to both internal and external stimuli [5,6].

### 3.1. Mechanisms of Protein Translation: Initiation, Elongation, and Termination

There are three key stages to the highly regulated protein translation process in eukaryotes: initiation, elongation, and termination.

Initiation is a crucial regulatory point that creates the foundation of protein translation. The 40S ribosomal subunit attaches to the eukaryotic initiation factors eIF1, eIF1A, eIF3, and eIF5 to make up the pre-initiation complex, enabling the start of initiation. The 40S subunit is then joined by the ternary complex, which consists of eIF2 coupled to GTP and Met-tRNAi (initiator methionyl-tRNA). The eIF4F complex, which consists of eIF4E (cap-binding protein), eIF4G (scaffold protein), and eIF4A (RNA helicase), recognizes the 5′ cap structure of mRNA and facilitates ribosome recruitment and mRNA unwinding. In an ideal Kozak context, the initiation complex searches the 5′ untranslated region (5′UTR) for the start codon AUG. Once it finds it, GTP is hydrolyzed, initiation factors separate, and the 60S large ribosomal subunit unites to form a functioning 80S ribosome that is prepared for elongation [36,37,38,39].

During elongation, the expanding polypeptide chain is gradually supplemented with amino acids. Eukaryotic elongation factors like eEF1A, transporting aminoacyl tRNAs to the A-site of ribosomes, and eEF2, which promotes ribosome translocation along the mRNA by a single codon, mediate peptide elongation. The integrity of this process is strictly upheld to prevent the addition of incorrect amino acids, which is essential for all cells, including neuronal proteins, whose function can be changed by even small sequence variations. Phosphorylation of these elongation factors in response to developmental cues or cellular stress can regulate elongation by modifying translation rates to satisfy cellular demands [36,39].

Finally, when the ribosome comes into contact with one of the three stop codons-UAA, UAG, or UGA, translational termination happens. To make the ribosome components available for subsequent translation cycles, release factors eRF1 and eRF3 identify these stop signals, mediate the release of the freshly produced polypeptide from the tRNA, and encourage ribosomal subunit separation. Any defect in this process can result in read-through mistakes or stalled ribosomes, which can affect neural development. Efficient termination is crucial for accurate protein synthesis [37,40,41].

### 3.2. Translation Regulation for Local Protein Synthesis in Neurons

The spatial separation between the nucleus, where gene transcription takes place, and distal synaptic locations, where translational activity occurs, is exclusive to neurons. Neurons deliver particular mRNAs to dendrites and axons in a translationally suppressed state to satisfy these spatial needs, allowing on-site protein translation in response to synaptogenesis.

Learning and memory processes are usually supported by this localized protein translation, which is essential for synapse formation, maintenance, and plasticity. Target mRNA transport and translational repression are controlled by RNA-binding proteins (RBPs), including zipcode-binding protein 1 (ZBP1), Staufen, and fragile X messenger ribonucleoprotein (FMRP). Upon brain activation, RBPs facilitate activity-dependent translational de-repression, which permits protein synthesis. To facilitate the local synthesis of proteins essential for synaptic modification, FMRP suppresses the translation of synaptic mRNAs until neural activation causes changes in its phosphorylation and release [42,43,44].

By altering the poly(A) tail length of mRNAs, the cytoplasmic polyadenylation element-binding protein (CPEB) family regulates translation and affects translational efficiency in a temporal manner that is closely related to neural activity and development. These local translational regulators are crucial since their disruption has been linked to neurodevelopmental disorders, such as fragile X syndrome (FXS) and autism spectrum disorders (ASD) [45,46].

Through promoter DNA methylation and histone modifications at specific genes, epigenetic mechanisms regulate the production of important neuronal RBPs, including FMRP and ZBP1, in FXS. Altered histone acetylation during neuronal differentiation regulates *ZBP1* levels, allowing activity-dependent mRNA delivery to dendrites. DNA methylation-dependent chromatin remodeling, which modifies poly (A) tailing, and temporal translation control at synapses, controls members of the CPEB family. The exact spatiotemporal expression of RBPs necessary for local protein synthesis is ensured by these epigenetic inputs [47,48].

### 3.3. Signaling Pathways Controlling Translational Machinery in Neurodevelopment

Protein synthesis is adapted to developmental and environmental stimuli by the convergence of many signaling pathways that control the start and elongation phases of translation.

**Figure 3:**
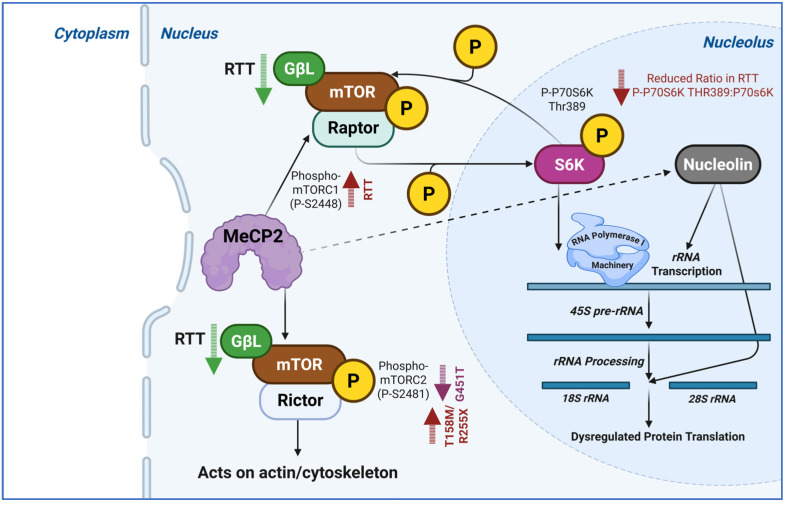
MeCP2-mediated modulation of nuclear mTOR signaling and nucleolar ribosomal biogenesis in Rett Syndrome. This schematic illustrates the regulatory role of MeCP2 in balancing the activity of nuclear mTORC1 and mTORC2 complexes and the subsequent impact on protein synthesis. In the nuclear compartment, MeCP2 influences mTOR signaling; however, Rett Syndrome (RTT) pathology is characterized by a significant increase in phospho-mTORC1 (P-S2448) and mutation-specific fluctuations in mTORC2 activity, such as the increase seen in T158/R255X variants versus the decrease in G451T, which may specifically disrupt actin and cytoskeletal organization. This dysregulation propagates into the nucleolus, where a reduced ratio of phosphorylated P70S6K to total P70S6K leads to the inhibition of the *ribosomal RNA* (*rRNA)* synthesis and nucleolin. Consequently, the transcription and processing of *45S pre-rRNA* into *18S* and *28S* subunits are impaired, culminating in the perturbed protein translation that underlies the neurodevelopmental features of the disorder. Figure adapted from [50] and generated by BioRender.

A key regulator of protein synthesis in neurons, the mammalian target of rapamycin complex 1 (mTORC1) integrates information from synaptic activity, growth factors (including BDNF), nutrition, and energy status (Figure 3). In addition to phosphorylating S6 kinase, which controls the synthesis of ribosomal proteins and translation factors, active mTORC1 also phosphorylates and deactivates eIF4E-binding proteins (4E-BPs), releasing eIF4E to start cap-dependent translation [49]. Developmental disorders such as focal cortical dysplasia and tuberous sclerosis complex are exacerbated by dysregulation of mTOR signaling, which leads to apparent neuronal growth and synaptic function involved in human disease [50,51,52]. Through the availability of acetyl-CoA and SAM (S-adenosyl methionine), mTORC1 also regulates DNA methylation and histone acetylation. PTEN (Phosphatase and tensin homolog deleted on chromosome 10) promoter hypermethylation maintains pathway activation, whereas nuclear mTORC1 attracts chromatin modifiers to ribosomal gene promoters [53,54].

Another important cellular pathway is the MAPK/ERK Pathway. By phosphorylating transcription factors and translation regulators, the mitogen-activated protein kinase/extracellular signal-regulated kinase (MAPK/ERK) cascade also regulates protein translation initiation, adjusting how neurons react to synaptic impulses and extracellular growth factors [55,56]. Histone acetyltransferases are drawn to immediate early gene promoters by ERK signaling. Neuronal reactions to synaptic inputs are modified by DNA methylation at route regulators [57].

Further regulation of protein translation is performed by a calcium-dependent mechanism. Synaptic activity and translation regulation are linked when intracellular calcium oscillations induced by neurotransmission activate calcium/calmodulin-dependent kinases (CaMKs), which phosphorylate translation components. This confirms the quick synthesis of new proteins at active synapses that are required for synaptic remodeling [58,59]. CaMKs recruit CBP/p300 for histone acetylation at neuronal-activity-regulated promoters via promoting CREB phosphorylation. For protein translation at synapses, activity-dependent DNA demethylation increases transcriptional output [57].

An additional signaling molecule is neurotrophin, involving molecules such as the BDNF. Neurotrophins, such as BDNF, activate downstream pathways like mTOR and ERK, which are essential for neuronal survival, differentiation, and synaptic plasticity via influencing translation through Trk receptors [60,61]. Of interest, *BDNF* is a downstream target gene of MeCP2 and controls the *MECP2*–*BDNF*–*miR132* regulatory feedback loop in the brain [18]. Trk signaling is regulated by activity-dependent DNA methylation at the *BDNF* promoter. Epigenetics and downstream translation are linked via MeCP2 binding to methylated *BDNF* regions [57].

### 3.4. Translational Dysregulation and Neurodevelopmental Disorders

Several neurodevelopmental and psychiatric problems are linked to the disturbance of the proper gene regulation in the brain. Loss of FMRP leads to excessive and uncontrolled translation of synaptic mRNAs, which is the primary hereditary cause of intellectual impairment in fragile X syndrome. Excessive protein synthesis may disrupt synaptic homeostasis, reducing cognitive function and plasticity.

Mutations in *TSC1* or *TSC2,* negative regulators of mTORC1, result in the tuberous sclerosis complex (TSC), which is characterized by benign tumors and neurological symptoms. Cognitive and epileptic characteristics can be explained by constitutive mTOR activation, which may cause apparent neural connections and excitability, as well as increased translation. Therapeutic rapamycin analogs that target mTOR pathways have demonstrated potential for symptom relief [62].

Additionally, leukodystrophy, affecting white matter development, is caused by mutations in translation initiation factors (eIF2B), underscoring the possible wider significance of translation machinery integrity in brain creation and maintenance [63].

The precise mRNA populations undergoing translation in different neuronal subtypes and at different phases of neurodevelopment are being revealed by recent developments like ribosome profiling and single-cell translational analysis. These methods possibly demonstrate that translation is controlled not only globally but also differently depending on the transcript and cell type. An intriguing new area that holds promise for understanding how neurons manage the many demands of development and experience-dependent plasticity is the dynamic interplay between epigenetic changes, connecting DNA methylation and translational control. To address the abnormal protein synthesis profile seen in brain disorders, treatment approaches that target translational regulators, mTOR modulators, or RNA-binding proteins are being developed, opening up new therapeutic options [6,64,65].

## 4. MeCP2 Is an Epigenetic Reader That Bridges DNA Methylation with Initiation of Protein Translation in the Brain

### 4.1. MeCP2 in Neurodevelopment

In the central nervous system, MeCP2 is a crucial epigenetic regulator that mainly reads DNA methylation marks, 5-mC and 5-hmC, controlling downstream gene expression programs that are essential for brain development. Post-mitotic neurons have higher levels of MeCP2 protein, which affects the transcriptional landscape by binding to methylated DNA and enlisting different coregulators that control histone modification states and chromatin compaction. The context-dependent dual function of MeCP2 is highlighted by its diverse activity, which goes beyond traditional transcriptional repression to encompass gene expression stimulation. MeCP2 plays a crucial role in preserving neuronal homeostasis and plasticity, and mutations in this protein cause RTT, marked by poor synaptic connections, altered neuronal maturation, and cognitive impairments. Key roles of MeCP2 in brain activity are further highlighted by its dose sensitivity [66,67,68,69]. Of note, MeCP2 has two main protein isoforms that are differentially expressed in the murine brain with some overlapping patterns, and their own expression is highly correlated with DNA methylation at the *Mecp2* regulatory elements [70,71,72,73,74].

### 4.2. Functional Consequences: Modulating Chromatin Accessibility and Synaptic Gene Networks

By interacting with repressor complexes such as histone deacetylases (HDACs) and the nucleosome remodeling and deacetylase (NuRD) complex, MeCP2 regulates chromatin accessibility by causing chromatin compaction and gene silencing at hypermethylated loci. On the other hand, MeCP2 can support activity-dependent synaptic gene expression by facilitating transcriptional activation through interaction with coactivators. MeCP2 can control the expression of genes essential for dendritic spine morphogenesis, neurotransmitter receptor modulation, and synaptic development, based on these diverse mechanisms of action. Furthermore, MeCP2 mediates the balance between neuronal excitation and inhibition, which is essential for learning and memory consolidation, and affects the epigenetic state of large genomic areas [75,76,77].

### 4.3. Ribosome Biogenesis and Protein Translation in Neurodevelopment

Protein synthesis, which is tightly regulated at different genomic levels, is especially demanding in neurons. Of note, mTOR signaling, ribosome biogenesis, and protein translation initiation are impaired in the brain of Rett Syndrome patients [50,51]. The mTOR signaling is one of the pathways that regulate ribosome biogenesis, a process that creates functioning ribosomes to translate transcripts into proteins. In order to regulate ribosome production and global translation rates, which in turn govern neuronal growth and synaptic plasticity, mTOR signaling combines external growth signals with intracellular nutritional status. MeCP2-deficiency has been connected to protein translation dysregulation and defective ribosome assembly in mouse models of RTT, affecting the synaptic proteome crucial for cognitive function. The intricate control of gene expression in the brain is demonstrated by the relationship between protein translation and epigenetic regulation, which ensures that protein synthesis is synchronized with developmental stimuli in both space and time [51,78].

### 4.4. Mechanistic Models and Unresolved Complexity of MeCP2 Function

**Figure 4:**
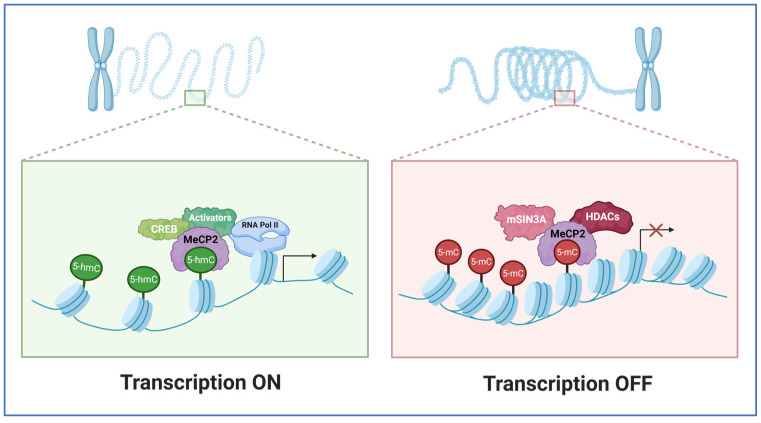
Regulatory role of MeCP2 in organizing chromatin architecture and gene expression. This figure illustrates the dual role of Methyl-CpG-binding protein 2 (MeCP2), which acts as a versatile transcriptional regulator by binding to methylated CpG sites to recruit corepressors like mSin3 and HDACs for transcriptional repression (red panel), directly facilitating nucleosome clustering for chromatin compaction, and associating with activators like CREB and RNA Polymerase II to promote transcriptional activation (green panel). Figure adapted from [84] and generated by BioRender.

A significant unanswered subject in the research is how MeCP2, as a single methylation DNA reader, can enable both transcriptional repression and activation in neurons. Beyond a basic concept of “on/off switch”, MeCP2 is a classical repressor that attracts SIN3A/HDAC or NCoR/SMRT complexes to methylated gene bodies or promoters, resulting in subsequent histone deacetylation and decreased transcription (Figure 4). According to a different perspective, MeCP2 is a global chromatin organizer whose extensive binding across methylated sequences (including mCA-rich regions) alters nucleosome dynamics and higher-order chromatin folding. In this model, transcriptional outcomes are dependent on local chromatin context rather than a fixed repressive function. MeCP2 post-transcriptional modifications, particularly phosphorylation at sites like S80, S165, S229, and neuronal activity-regulated S421, are highlighted by a third line of evidence. Although these phospho-specific interactions offer a tenable explanation for context-dependent function, it is still unclear how they affect RBPs and other initiation factors. These findings collectively support an explicitly dynamic MeCP2 paradigm, in which transcriptional dampening, buffering, or facilitation of specific loci is concurrently determined by DNA methylation density, 5-hmC versus 5-mC abundance, chromatin state, and MeCP2 modification status [79,80,81,82,83].

Non-CpG (CpH/mCH) methylation and its function in the interplay between epigenetics and protein translation interface represent another complex mechanism. During postnatal brain maturation, neurons experience remarkably high quantities of mCH, enriched at the gene bodies rather than promoters, and inversely linked with transcription at many lengthy neuronal genes. The expression of transcripts linked to MeCP2-related disorders is altered when MeCP2 binds mCH, especially mCA, at these lengthy genes. Genome-wide data suggests that mCH can act independently of mCG and may correlate with active transcription in certain contexts, such as escape genes on the inactive X chromosome [79,80,85,86,87].

## 5. Integrative Mechanisms and Future Directions

### 5.1. Linking DNA Methylation to Protein Translation

DNA methylation and initiation of protein translation have a complex interplay that may include both direct effects on gene transcription or indirect modulation through epigenetic reader proteins that may affect signaling molecules upstream of protein translation. The chromatin architecture is in part influenced by DNA methylation patterns, which in turn regulate gene transcription that would be subject to subsequent protein translation. Proteins such as MeCP2 may control translational regulators, which may impact initiation factors and ribosome recruitment. Through the coordination of brain progenitor differentiation, synapse formation, and crucial phases of plasticity, this multilayer regulation supports the claim that neurodevelopment progresses with precisely determined timing and regional uniqueness. Studies have started to identify feedback loops in which translational output can affect the epigenomic landscapes, indicating a dynamic interaction sensitive to neuronal and environmental activity [9,88,89,90].

The reciprocal control of DNA methyltransferases and translational signaling pathways is one molecular level at which DNA methylation and protein production may interact. A feed-forward loop, in which growth-factor-driven protein translation directly remodels the methylome, is seen in hepatocellular carcinoma, where hyperacetylation of mTORC1 increases the translation of DNMT1 in a 4E-BP1-dependent way, increasing global DNA methylation. By stabilizing oncogenic DNA methylation patterns, the mTOR-DNMT1 axis creates a causal model that reinforces translational upregulation by silencing negative regulators of mTOR signaling and other tumor suppressors [91].

By regulating the expression of essential elements of the translational machinery and their epigenetic regulators, DNA methylation also functions upstream of protein translation. In mammary epithelial cells, hypomethylation and chromatin relaxation at the *ribosomal DNA (rDNA)* gene causes, for instance, by the loss of the histone demethylase JHDM1B, an increase in *45S pre-rRNA* transcription and processing, increasing ribosome biogenesis and translational output. The expression of translation initiation factors can also be modulated by promoter methylation. In prostate cancer, a reduced level of DNA methylation at the *eIF4A1* locus leads to its increased transcript and protein levels, contributing to elevated activity of the eIF4F complex, which regulates cap-dependent translation of carcinogenic mRNAs [92,93,94].

DNA methylation-dependent regulation of RNA modification pathways, which directly adjust translational efficiency, is another layer of interaction. In order to decrease mRNA, m6A (N6-methyladenosine) deposition and the translation efficiency of particular targets, like *TOP2B*, DNA methylation at the *METTL14* promoter can limit SP1 binding and downregulate *METTL14* transcription. These effects are then selectively amplified when DNA methylation is relaxed. This creates a regulatory loop whereby m6A status influences mRNA stability and ribosome loading, DNA methylation patterns determine the abundance of m6A writers, and the ensuing modifications in protein synthesis influence epigenetic states, through altered expression of chromatin, related genes, and DNA methylation regulators [57,95,96].

### 5.2. Emerging Techniques and Perspectives

Our comprehension of the interplay between translational control and DNA methylation has been transformed by recent developments in multi-dimensional genomics. These days, single-cell multi-omics techniques allow the simultaneous assessment of protein synthesis, RNA expression, and DNA methylation within individual cells, producing elaborate maps of neurodevelopmental trajectories that are specific to certain cell types. By placing this molecular process inside the three-dimensional tissue architecture, spatial transcriptomics and epigenomics provide an additional vision for comprehending regional- and cell-type-specific development in the brain. Ribosome profiling techniques provide snapshots of translation dynamics, allowing cause-and-effect investigations that could address mechanistic ambiguities, while CRISPR-based epigenome editing technologies increasingly enable precision analysis of DNA methylation at target loci. When combined, these methods have the capacity to transform the understanding of the molecular reasoning behind brain growth and disease state [97,98,99,100,101]. High-resolution maps of DNA methylation at genomic loci encoding translation factors (eIF4E, RPS6) are correlated with ribosome occupancy on synaptic mRNAs shown by single-cell multi-omics platforms that simultaneously profile DNA methylation (using enzymatic conversion or bisulfite sequencing) and ribosome profiling (Ribo-seq). This indicates how cell type-specific DNA methylation represses RBP expression to calibrate local translational capacity during neuronal differentiation and circuit maturation. DNA methylation dynamics are causally linked to synapse-specific translational control and plasticity defects in neurodevelopmental disorders. Similarly, CRISPR-dCas9 (dead Cas9) epigenome editing tools, where dCas9 is fused to DNMT3A or TET1, when combined with spatial transcriptomic techniques (i.e., MERFISH or Slide-seq), enable precise changes in DNA methylation at promoters of RBPs (i.e., *FMRP* or *ZBP1*). Such techniques can concurrently visualize downstream alterations in transcript localization, polyadenylation, and protein synthesis hotspots within intact brain tissue slices. Nucleosome occupancy and methylome sequencing (NOME-seq) combined with single-nucleus multiome assays (scATAC-seq for chromatin accessibility and scRNA-seq for transcriptome) analyze methylation-dependent changes in enhancer accessibility at *rDNA* repeats and translation regulator genes, identifying epigenetic bottlenecks that limit ribosome biogenesis and polysome formation in different neuronal subtypes in the brain. Lastly, perturb-seq methods distinguish direct from indirect effects of methylation on mRNA stability, epitranscriptomic marks (m6A), and polysome association by using CRISPR interference (CRISPRi) against methylation readers like MeCP2 in conjunction with scRNA-seq. This helps to elucidate reader-specific mechanisms of translational repression and de-repression in response to synaptic activity. In addition to resolving long-standing mechanistic difficulties, the combination of these approaches opens the door to analyzing the casual epigenomic contributions to brain development and disease [79,80,85,86,87].

### 5.3. Therapeutic Implications

Currently, most neurodevelopmental disorders have no cure. Therefore, therapeutic approaches to address translational and epigenetic dysfunctions have gained attention. In an attempt to restore balanced gene expression and reverse abnormal developmental trajectories, pharmacological treatments that target DNA methyltransferases and histone-modifying enzymes are studied. Complementary strategies that target translational machinery, including modulators of mTOR signaling, provide ways to restore normal amounts of protein synthesis, improving synaptic function and cognitive outcomes. In preclinical studies, gene therapy to restore functional MeCP2 protein that supports translation fidelity and ribosome biogenesis shows promise in the context of RTT. There is also current research with metabolic drugs (such as metformin and statins) indicating potential beneficial effects for RTT using mouse models [102,103,104,105]. For neurodevelopment and neuropsychiatric illnesses, further investigation of epigenetic translational interaction may provide new targets and combinatorial treatments [102,106,107,108,109,110].

Although it is theoretically appealing to target DNA methylation with pharmaceuticals, existing hypomethylating drugs highlight the difficulty of accomplishing cell-type- and locus-specific editing in the brain. Nucleotide DNMT inhibitors, like 5-azacytidine and decitabine, incorporate into DNA and cause widespread hypomethylation across repetitive elements and gene promoters. Such an approach may be applicable in myelodysplastic or leukemic cells that divide quickly, but it may cause problems in post-mitotic neurons that rely on stable DNA methylation to preserve identity and circuit integrity. Concerns include unintentional reactivation of proto-oncogenes, genomic instability at satellite repeats, and associated subsequent transcriptional reprogramming in non-target tissues or cells. Accordingly, before brain-directed epigenetic therapies can be safely implemented, there are still unanswered questions regarding specificity (isoform-specific DNMT1/3A/3B inhibition), administration (brain-penetrant small molecules, nanoparticles, or conjugates), and consequences in differentiated neurons [111,112,113,114].

Similarly to this, therapies targeting mTOR signaling show both therapeutic limitations and promise. In tuberous sclerosis complexes, rapamycin and its analogs reduce seizures, tumor burden, and occasionally cognition, indicating that in certain hereditary settings, suppressing overactive mTORC1 can be helpful. However, strong inhibition of mTORC1 can prevent late-phase long-term potentiation (LTP) and long-term memory (LTM) formation, according to pharmacogenetic and pharmacological research, suggesting that long-term suppression of this pathway may compromise synaptic plasticity in otherwise healthy neuronal circuits. In addition to having systematic drawbacks like immunosuppression and metabolic adverse effects, chronic mTOR regulation may function as a variable switch rather than a straightforward on/off switch for plasticity. These findings draw attention to a key therapeutic dilemma: how to determine dosage schedules, brain penetration, and potentially cell-type restricted interventions that correct pathological mTOR hyperactivation without overshooting into harmful hypoactivity in neurons necessary for normal learning and development [115,116].

### 5.4. Gaps in Knowledge

Despite rapid progress, certain fundamental components of the DNA methylation-translation interface in the brain remain either poorly understood or subject to contradictory evidence. The primary function of neuronal DNA methylation and MeCP2 still remains a dogma. While some studies support a model in which MeCP2 globally regulates expression of long, highly methylated genes, quantitative analyses argue that DNA methylation and MeCP2 explain only a small fraction of transcriptional or splicing variation, challenging a straightforward concept of the “global repressor” or “splicing regulator” models. Similarly, new research demonstrates that mCA and MeCP2 sustain long-gene expression programs particular to neuron types; however, it is still entirely unclear how cell type-specific mCA patterns are decoded at enhancers and promoters to shape translationally important genes [79,117,118,119].

The relationship between translational control and the epigenetic “timers” of neuronal maturation represents another gap in knowledge. There is evidence that translation of chromatin regulators itself can tune fate specification, and epigenetic barriers involving EZH2, DOT1L, and EHMT1/2 cells may control species- and cell type-specific maturation rates. Nevertheless, we lack fully comprehensive integrated models that quantitatively link difficult chromatin structure and function to activity-dependent protein translation and proteome remodeling in defined neuronal circuits [117,120]. Despite intensive efforts, there are a lot of questions that still need to be answered.

## 6. Conclusions

A key component of brain development is the interaction between DNA methylation and protein translation, which coordinates precise gene expression programs that, in the brain, control synaptic connections, neural progenitor differentiation, neuronal maturation and synaptic plasticity. When compared to other organs, the brain has a unique DNA methylation landscape (about 70% of the genome is methylated) in terms of both the total amounts and the genomic distribution of modified cytosines. Neuronal tissues exhibit highly tissue-specific differentially methylated regions close to neurodevelopmental genes that are not modified, similarly to peripheral tissues such as blood. The predominance of non-CG (mCH) methylation and relatively high levels of 5-hmC in neurons, which are uncommon or significantly lower in the majority of other cell types, are characteristics of the brain and neurons. These features are functionally linked to the control of neuronal genes expressed through the functioning of epigenetic readers like MeCP2. While regulation of protein translation fine-tunes the final proteomic landscape that would support brain function, epigenetic modulation through DNA methylation lays the groundwork for the chromatin states. MeCP2, a complex molecule whose alteration seriously impairs brain development and function, serves as an example of the crucial connection between these processes. Modern technological advancements in translational profiling and single-cell epigenomics have shed light on the complex molecular action underlying general development and have raised the prospect of potential treatment approaches that target the underlying cause of brain diseases. The combination of translational regulation and epigenetics opens up exciting new avenues for the study and treatment of neurodevelopmental disorders.

Despite intensive progress, there are still a number of significant unanswered concerns about the mechanistic integration of epigenetics and protein translation in the developing and adult brain. The question of whether DNA methylation and its readers serve mainly as slow stabilizers of neuronal gene expression and translational programs or as quick, switch-like regulators that can quickly alter local protein synthesis in response to stimuli from the environment or activity remains unanswered. According to competing hypotheses, MeCP2 either imposes more context-dependent, gene-specific control that is closely linked to certain genetic networks and cell types, or it broadly scales the transcription and downstream translation of long, methylation-rich neuronal genes. Additionally, it is still unknown how the translation of epigenetic regulators interacts with epigenetic “barrier” mechanisms that determine the timing of neuronal maturation through chromatin modifiers like EZH2, DOT1L, and EHMT1/2, and whether these two layers function in tandem or as a part of a single temporal control system. Determining how much of the DNA methylation-protein translation interplay is species-specific versus conserved across brain regions and neuronal lineages, as well as how such diversity affects the resilience or susceptibility of different circuits in neurodevelopmental and neuropsychiatric disorders, remains an unresolved challenge.

## Data Availability

Data sharing is not applicable. No new data were created or analyzed in this study.

## Abbreviations

The following abbreviations are used in this manuscript:

5-hmC	5-hydroxymethylcytosine
5-mC	5-methylcytosine
ASD	Autism Spectrum Disorders
*Bdnf*	Brain-derived neurotrophic factor
CaMKs	Calcium/calmodulin-dependent Kinases
CPEB	Cytoplasmic Polyadenylation Element-Binding protein
*Dali*	*DNMT1-associated long intergenic RNA*
DNMT1, DNMT3A, DNMT3B	DNA Methyltransferases 1, 3A, and 3B
FMRP	Fragile X Messenger Ribonucleoprotein
HDACs	Histone Deacetylases
LTM	long-term memory
LTP	long-term potentiation
MBD	Methyl-CpG-binding domain
MeCP2	Methyl-CpG-binding protein 2
mTOR	mammalian Target of Rapamycin
NuRD	Nucleosome Remodeling and Deacetylase complex
RBPs	RNA-binding proteins
*rDNA*	*ribosomal DNA*
*rRNA*	*ribosomal RNA*
RTT	Rett Syndrome
SET-domain	Su(var)3-9, Enhancer-of-zeste and Trithorax domain
TET	Ten-Eleven Translocation
TSC	Tuberous Sclerosis Complex
ZBP1	Zipcode-binding protein 1
